# Vole hunting: novel predatory and carnivorous behavior by California ground squirrels

**DOI:** 10.1007/s10164-024-00832-6

**Published:** 2024-12-18

**Authors:** Jennifer E. Smith, Joey E. Ingbretson, Mackenzie M. Miner, Ella C. Oestreicher, Mari L. Podas, Tia A. Ravara, Lupin M. L. Teles, Jada C. Wahl, Lucy M. Todd, Sonja Wild

**Affiliations:** 1 Department of Biology, University of Wisconsin – Eau Claire, Eau Claire, Wisconsin 54701 USA; 2 Department of Environmental Science and Policy, University of California, Davis, CA 95616 USA; 3 Institute of Biological Sciences and Health, Federal University of Alagoas, Maceió, Alagoas, 57072-900 Brazil

**Keywords:** Hunting behavior, Predator–prey interaction, Sciuridae

## Abstract

Dietary flexibility allows animals to respond adaptively to food pulses in the environment. Here we document the novel emergence of widespread hunting of California voles and carnivorous feeding behavior by California ground squirrels. Over two months in the twelfth year of a long-term study on the squirrel population, we document 74 events of juvenile and adult ground squirrels of both sexes depredating, consuming, and/or competing over vole prey. Our video footage, photographic evidence, and direct observations of marked individual squirrels provide insights into the ecological circumstances favoring behavioral flexibility in foraging associated with a decadal peak in vole abundance. Digital video images related to the article are available at http://www.momo-p.com/showdetail-e.php?movieid=momo241126ob01a.

**Supplementary Information:**

The online version contains supplementary material available at 10.1007/s10164-024-00832-6.

## Introduction

Behavioral flexibility is an important mechanism by which animals may respond to changing environments (Wright et al. 2010). Within the context of foraging, dietary shifts allow animals to flexibly respond to changes in foraging risks and opportunities (Abrams 2010). Indeed, a large literature exists on the ‘ecology of fear’, demonstrating that many prey species, including various squirrel species (family Sciuridae), dynamically adjust their foraging decisions to predation risk (e.g., Brown et al. 1999; Palmer et al. 2022; Ortiz-Jimenez et al. 2025). However, far fewer studies have systematically documented the behavioral shifts of squirrels to large pulses of food, and most focus on the anticipation of seeds associated with masting events (e.g., Boutin et al. 2006). This is likely because the diet of squirrels is made up of mainly acorns, seeds, nuts, and fruits (Thorington et al. 2012). Any supplementation of their vegetarian diet was historically believed to primarily occur through eating insects or, on occasion, nest predation of eggs or young hatchlings (Bradley and Marzluff 2003).

Roughly 30 years ago, Callahan (1993) radically altered our perception of squirrels by characterizing as many as 30 species of the family Sciuridae as facultative predators of small vertebrates capable of killing and consuming adult fish, amphibians, reptiles, birds, and mammals (Table S1). Despite the growing consensus that many squirrel species opportunistically consume meat (Callahan 1993; O’Donoghue 1994), much of the early evidence for predation is based on stomach contents or the killing of heterospecifics in captive settings (e.g., zoos, traps). This makes it challenging to distinguish between scavenging and direct predation. The direct study of hunting behavior by squirrels remains rare, and most reports in field settings are still limited to a single depredation event (Table S1). Studies characterizing the demographic and social aspects of hunting behavior in free-living squirrels are therefore needed to better understand these patterns and inform our understanding of the processes shaping hunting behavior in mammals more broadly. Such studies also offer important insights into behavioral flexibility.

Here, we provide the first evidence of California ground squirrels (*Otospermophilus beecheyi*, Richardson, 1829, formerly Beechey ground squirrel, and recently distinguished from *O. douglasii* (Smith et al. 2016; Long and Smith 2023)) repeatedly hunting, killing and consuming adult vertebrate prey in nature. Native to California grasslands, this ecosystem engineer is a major prey species for mammalian carnivores, snakes, and birds (Smith et al. 2016). It is a socially tolerant species that resides in groups structured by fission–fusion dynamics (Gall et al. 2022; Person et al. 2024). It typically forages alone or in small groups (Ortiz et al. 2019; Owings et al. 1977) on seeds from grasses and oaks (Linsdale 1946), and almost exclusively on green herbaceous vegetation in the growing season (Fitch 1948). It consumes leaves, flowers, buds, stems, shoots, roots, tubers, twigs, and bark (Grinnell and Dixon 1918; Evans and Holdenried 1943; Linsdale 1946; Fitch 1948) from over 100 different native and invasive plant species (Smith et al. 2016) and is a serious agricultural pest of fruit, nut, and vegetable harvests (Grinnell and Dixon 1918; Stanton 1944; Baker 1984; Schramm and Bullard 2004).

There is some evidence of meat-eating in California ground squirrels. Despite its primarily granivorous diet, previous studies have documented occasional consumption of invertebrates (Evans and Holdenried 1943; Stanton 1944; Baker 1984; Carlton and Hodder 2003) and avian eggs or nestlings of killdeer, California quail, bobwhite quail, ring-necked pheasant*,* mourning dove, dark-eyed junco, and American robin (Grinnell and Storer 1924; Emlen and Glading 1938; Stanton 1944; Fitch 1948; Linsdale 1946; Leopold 1977; Baker 1984; Purcell and Verner 1999; Yeh et al. 2007Y; Bataille and Baldassarre 1993). It has also been reported to eat eggs of domestic chickens (Grinnell and Dixon 1918; Howell 1938) and a small fish, the California grunion (Olson 1950). Fitch (1948) observed it consuming, but not directly killing, young desert cottontails, adult pocket gophers, and kangaroo rats.

Several studies have also reported scavenging of trapped fish, meat, woodrats (*Neotoma*), songbirds, and, in one case, an adult conspecific (Grinnell and Dixon 1918; Fitch 1948; Miller and Stebbins 1964). It also has killed, but did not eat, young gopher snakes (Fitch 1948). The direct killing of adult animals is limited to a few reports such as the killing and eating of side-blotched and western fence lizards in captivity (Sandberg and Banta 1973), a possible killing of a domestic chicken (Grinnell and Storer 1924), and the killing and consumption of several Northern broad-footed moles (Trulio et al. 1986). Infanticide, the direct killing of newly emerged young has also been documented extensively in California ground squirrels (Trulio et al. 1986; Trulio 1996). For example, 40 killings of post-emergent juvenile ground squirrels were documented over 4 years; juveniles were cannibalized in at least 22 of these cases (Trulio 1996).

**Fig. 1:**
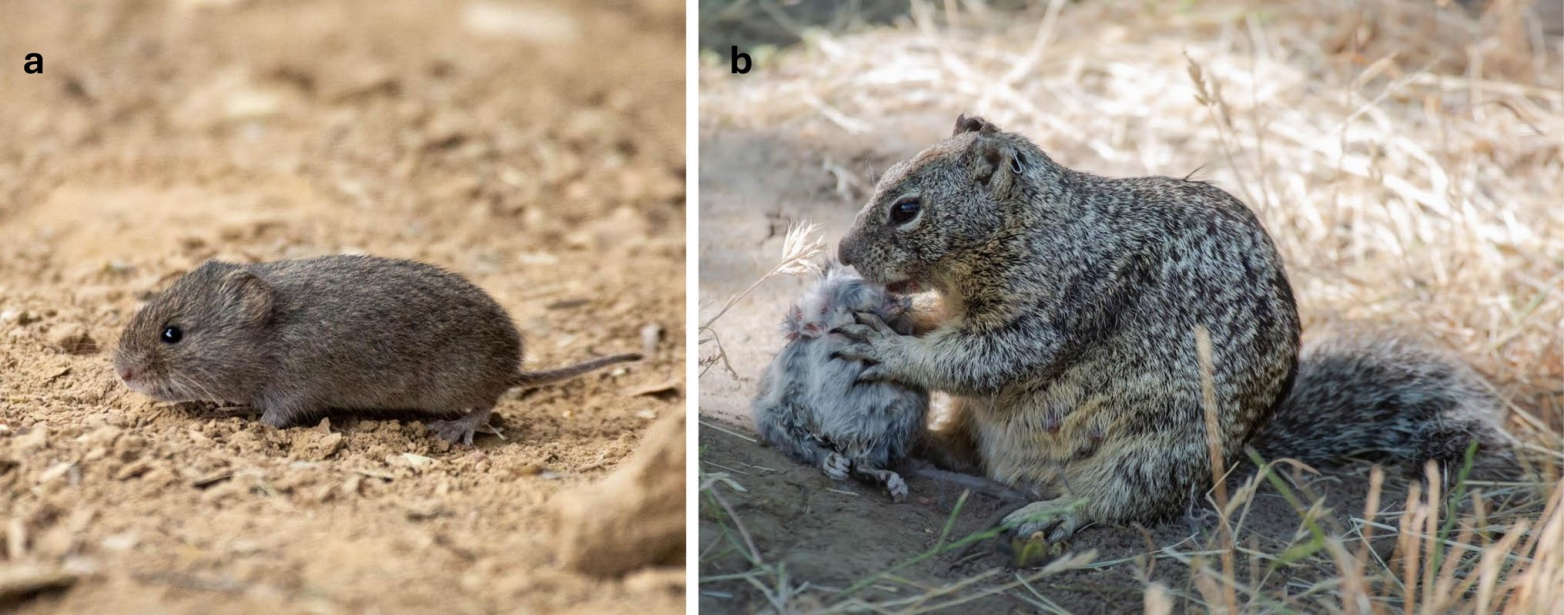
**a** An adult California vole at the study site and **b** an adult female California ground squirrel consuming the head of a freshly hunted adult California vole (http://www.momo-p.com/showdetail-e.php?movieid=momo241126ob01a)

For the first time, we document the occurrence of widespread hunting of mammalian prey, the California vole (*Microtus c. californicus*) by the California ground squirrel (Fig. 1; http://www.momo-p.com/showdetail-e.php?movieid=momo241126ob01a; SI). The California vole is a species that feeds primarily on grasses and sedges and is mainly preyed upon by hawks, owls, egrets, long-tailed weasels, coyotes, skunks, mountain lions, and garter snakes (Cudworth and Koprowski 2010). Although some population densities are stable (Ostfeld and Klosterman 1986), most cycle, peaking every 3–5 years (Garsd and Howard 1982) at densities of up to roughly 400–2000 voles per hectare (Pearson 1966; Batzli and Pitelka 1971; Lidicker 1973; Ostfeld et al. 1985; Cudworth and Koprowski 2010) with mean rates of increase from 0.08 to 2.14 in open areas (Lidicker and Anderson 1962). In 2024, parts of Northern California experienced vole ‘infestations’ (Curry 2024; Hill 2024) and our team also noticed more voles at the study site compared to any of the previous years of our long-term study. After a decadal peak in numbers of California voles (hereafter “voles”), and in the twelfth year of study on California ground squirrels (hereafter “ground squirrels”), we document the emergence and widespread nature of the newly observed hunting of voles by ground squirrels. We characterize the demographic and social aspects of this novel behavior never previously observed in our study population. We discuss how these observations fundamentally change our understanding of the dietary flexibility of this ground squirrel.

## Methods

### Study site and subjects

**Fig. 2:**
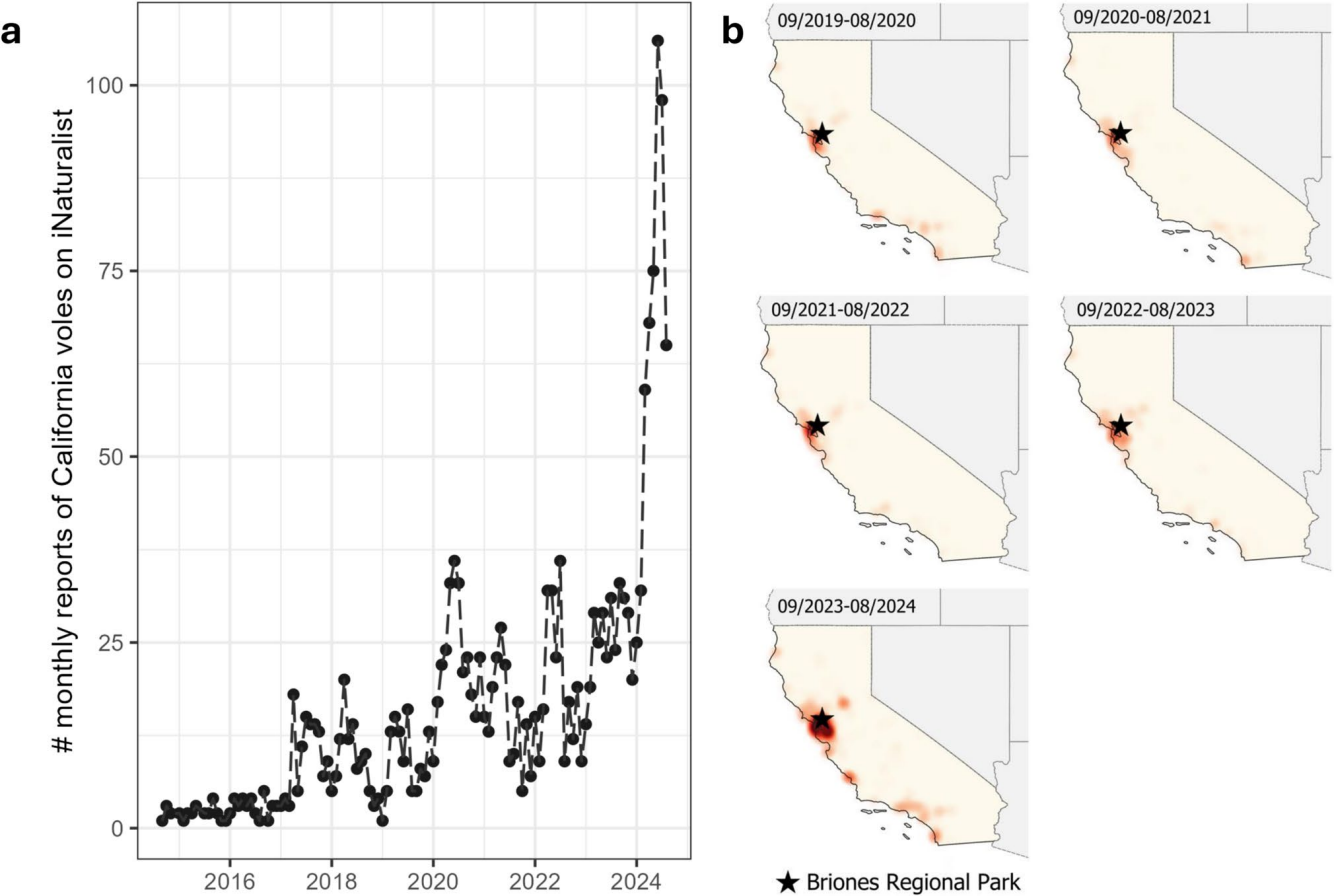
Number of monthly reports of California voles across **a** time (10 years: September 2014–August 2024) across the state of California, and **b** geographic location (5 years: September 2019–August 2024)

The current research is part of a long-term study on the behavioral ecology of California ground squirrels in a 9596 m^2^ recreational area with open grassland, walnut, and oak trees at Briones Regional Park in Contra Costa County, California in the United States (37.9377014 N, 122.1388542 W, WGS 84; Fig. 2b; Hammond et al. 2019). Since 2013, we have live-trapped, marked, and released known individuals in June and July, the time of year when most animals, including young of the year, are active aboveground (Tomich 1962; Smith et al. 2018). On a biweekly basis, Tomahawk live traps were baited with sunflower seeds and peanut butter. Sex, body mass, and reproductive status of each individual were noted. Upon first capture, we marked each individual with a uniquely numbered Monel metal ear tag (National Band and Tag Co.) and a passive integrated transponder (PIT) tag (Biomark, Inc.). For visual identification during observations, we also painted a unique fur mark on their backs using Nyanzol dye (Greenville Colorants).

While we did not directly quantify vole densities at our field site, we extracted reports of California voles from the citizen science platform iNaturalist (iNaturalist community 2024) between September 2014 and August 2024 at this site and other regions across the state of California, and compared the highest peak in 2024 to the number of reports in previous years.

From June 10th to July 30th 2024, we recorded all sightings of squirrels hunting, killing and/or consuming voles both opportunistically on trapping days and during formal behavioral observations. Formal behavioral observations were conducted on non-trapping days between 0800 and 1200 h on a total of 16 weekdays between June 7th and July 25th 2024. To ensure full coverage of the study site, three groups of observers watched subsets of the population at the same time; each group sat ≥ 20 m from squirrels to minimize disturbance of the subjects (Gall et al. 2022). Each set of observers was responsible for a spatially distinct area at the study site and communicated via radios during observations to avoid any potential overlap in instances reported here. Combined notes were integrated into the final data set after being thoroughly checked to resolve any potential redundancies in the data collection. Areas visible to each set of observers were watched for 51, 45, and 50 h, respectively, for a total of 146 observation hours.

During formal observations, we recorded all occurrences of vole hunting, killing and/or consumption as well as all occurrences of social interactions among conspecifics (Altmann 1974). Starting times and locations of these behavior were logged relative to the nearest natural (e.g., tree) or artificial (e.g., picnic table) landmarks in the study area (for details, see Ortiz-Jimenez et al. 2022). Affiliative behaviors included greeting, sitting, or foraging in close proximity (< 1 m), and play, whereas intraspecific competition was characterized by agonistic behaviors (e.g., displacement, chases, and physical pushing, pouncing, or biting; Smith et al. 2016).

### Statistical analyses

To explore the demographic aspects of this novel behavior, we applied three separate Fisher’s exact tests comparing the number of adult females, adult males, juvenile females and juvenile males engaging in hunting of, consumption of, and competition over voles (versus those that did not) in the whole population. To ensure sufficient opportunities to observe these behaviors, analyses were limited to marked individuals that were present on at least three formal observation days.

## Results

### A peak in vole abundance

Overall, vole sightings reported on iNaturalist across California fluctuated over time, but numbers reported in 2024 far exceeded numbers from the past decade (Fig. 2a). In 2024, the peak in vole abundance was roughly seven times greater than the 10 year average. Over these ten years, reports of vole sightings were also higher at Briones Regional Park than those reported across the state (Fig. 2b).

### Occurrences of vole events during field season

**Fig. 3:**
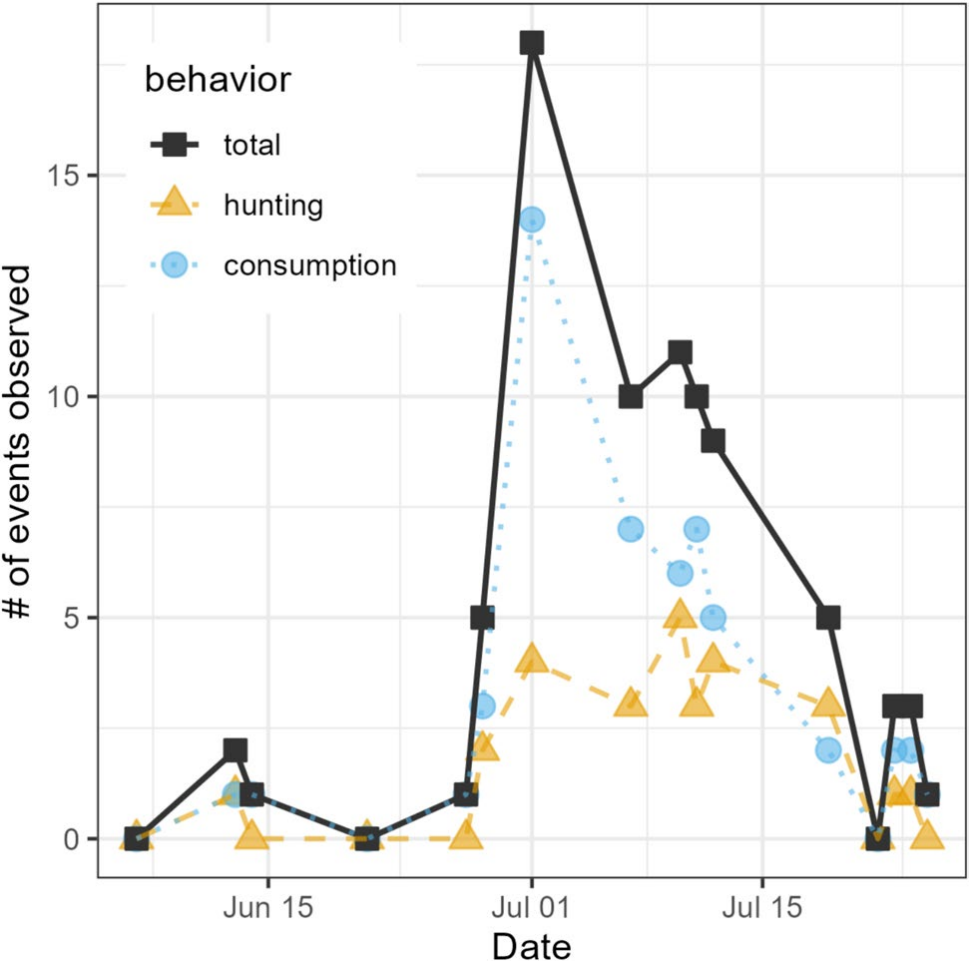
Number of observations of vole hunting and consumption (and total number of vole interactions) observed on formal observation days

We recorded a total of 74 events of vole hunting and/or consumption over a total of 18 days of fieldwork (Fig. 3a). Sixty-five of these events occurred on formal observation days (Table S2). We observed vole hunting and/or consumption on nearly all (*N* = 13 days, 86%) of the 16 formal observations days during the season, with incidents peaking during the first two weeks of July (Fig. 3). The identity of foraging squirrels was clearly recognizable for 51 (69%) of the 74 events, involving a total of 27 unique squirrels. Across the field season, we observed a total of 125 marked individual squirrels from the study population during social observations, of which 53 marked squirrels were observed on three or more different observation days. Of the 27 identified squirrels seen interacting with voles, 26 (96%) were observed on at least three observation days and therefore included in our statistical analyses (Table S2).

### Behavioral patterns associated with hunting and consumption

Out of 74 events of observed interactions with voles, nearly half of these (*N* = 31 events, 42%) involved active hunting of voles by ground squirrels (Table S2). *We define hunting as the active pursuit of prey.* For three hunting attempts, we noted squirrels staying low on the ground and minimizing noise (stalking) before launching an attack. Nineteen hunting attempts involved a ground squirrel chasing a single vole across the landscape. If pursuit allowed squirrels to come sufficiently near prey, it was usually followed by a pounce on the prey to restrain it with forepaws and teeth. Killing attempts typically involved one or several bites targeting the neck area of the vole (but often also other body parts) with vigorous lateral bite-shaking of prey noted on one occasion. Hunters successfully captured and killed a vole in 17 of the 31 observed hunting attempts (55%). The other 14 attempts failed; prey either got away during pursuit or escaped after being initially captured by a squirrel. Squirrels rarely employed a sit-and-wait strategy of stalking and ambushing prey from a motionless position while hidden in tall grass. Instead, hunting attempts were best characterized by squirrels opportunistically chasing a single vole over a short distance in open areas, across dirt substrate. Although successful hunting was sometimes followed by direct consumption of the vole at the site of capture, a majority of events (70%, 12 out 17 kills) involved a squirrel carrying the dead vole in the mouth to a different location or, less often, out of sight to a burrow refuge. In all eleven of the events for which consumption of an intact carcass was observed, squirrels first removed the head of the vole (Fig. 1b). Next, they either directly pulled meat out of the torso or first stripped fur from each of the body parts before consuming the exposed meat, organs, and cartilage (see online video footage).

### Demographic patterns of vole hunting and consumption

**Fig. 4:**
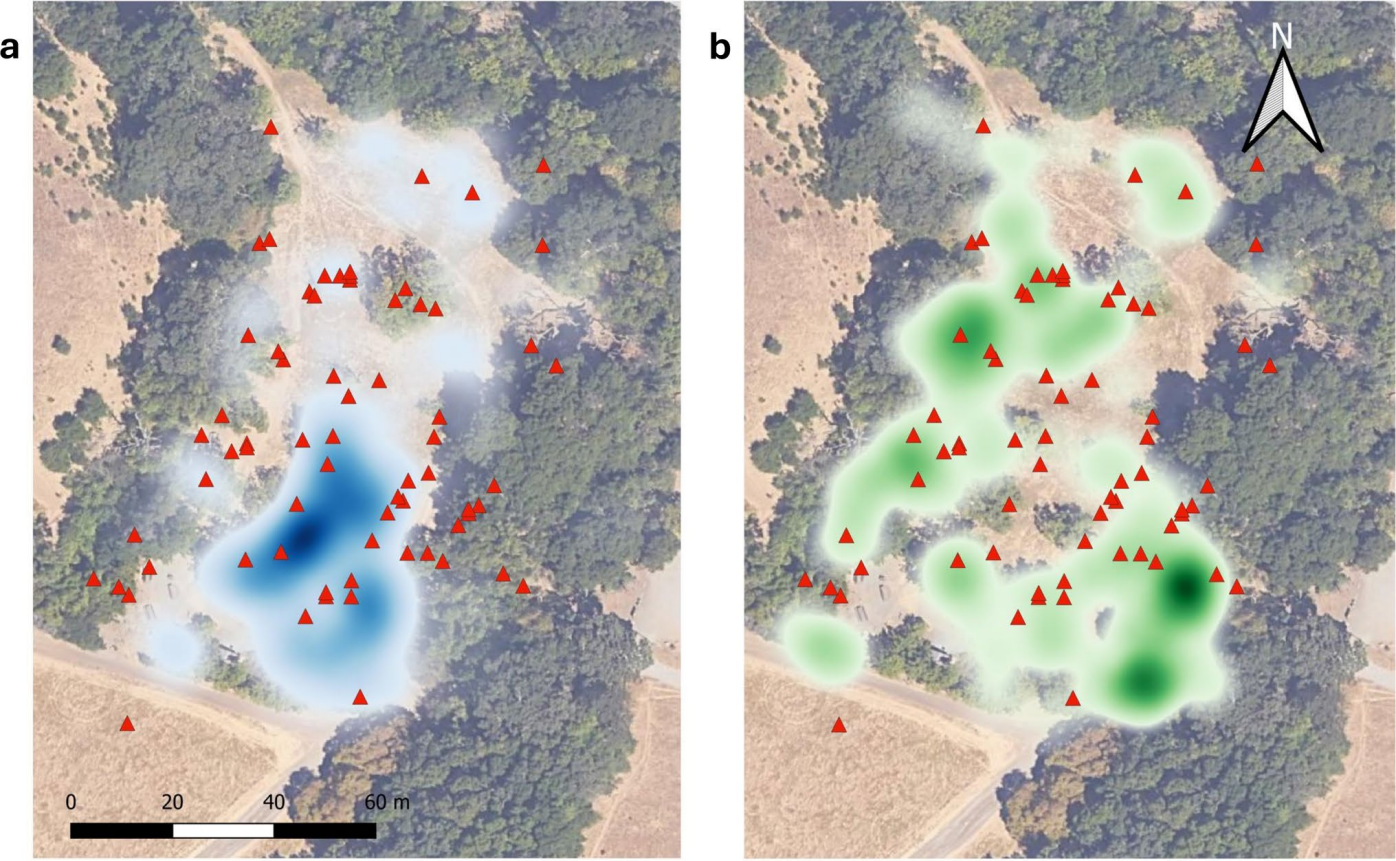
Map of study area overlaid with heat map of **a** vole burrow density in blue and** b** squirrel density in green (darker shades = increased density), and locations of vole hunting and consumption (red triangles). Aerial imagery of study site © Google, Map data © 2024

Overall, participation in hunting and consumption of voles was widespread across members of the study population and across the study area (Fig. 4; Table S2). Rather than being concentrated at vole burrow locations (Fig. 4a), ground squirrel–vole interactions generally occurred in areas of high ground squirrel densities, especially in open areas with low ground and tree cover (Fig. 4b).

**Fig. 5:**
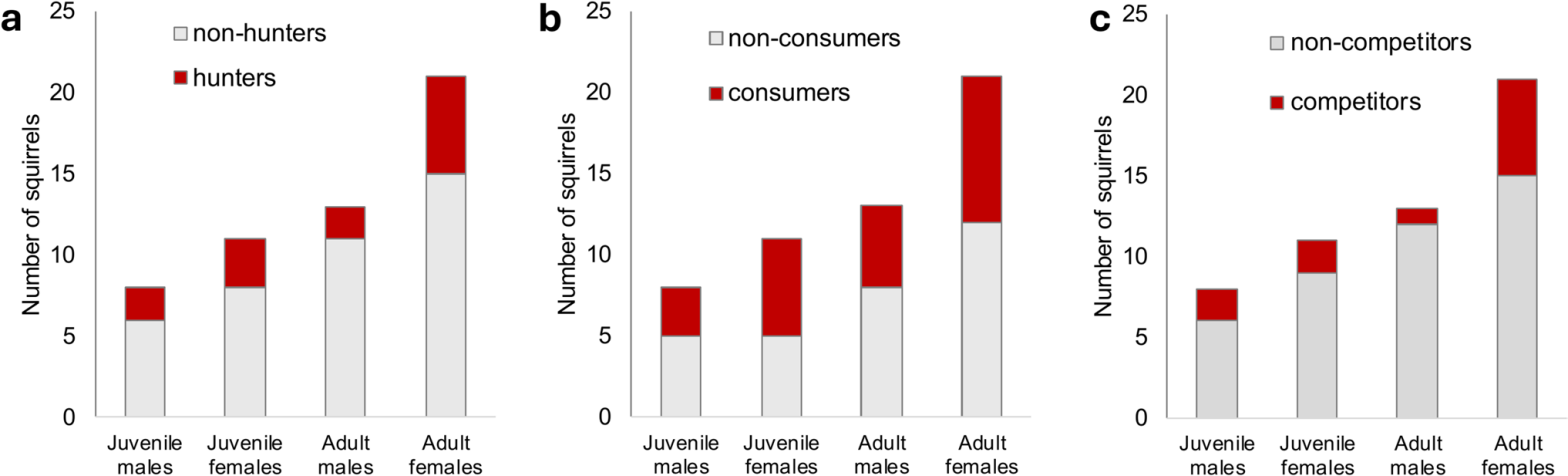
No effect of age–sex category of California ground squirrels on their participation in **a** hunting of, **b** consumption of, and **c** competition over California voles

Age and sex of hunters (*p* = 0.87, Fig. 5a), and consumers (*p* = 0.92, Fig. 5b) did not predict their participation in these activities relative to the background population. Adults were successful in 10 out of 17 hunting attempts (59%), while juveniles were successful in 7 out of 14 hunting attempts (50%). The precise identity of the hunter was discerned for 22 of all (attempted) hunting events; the remaining hunts were by 5 unidentified juveniles and 4 unidentified adults. In total, adult females were responsible for roughly half of these hunting events. Specifically, we identified a total of 2 juvenile males (15%), 3 juvenile females (23%), 2 adult males (15%) and 6 adult females (46%) from the more tractable hunting events with individuals being observed engaging in hunting between one and four times (Table S2). Most hunting events involved solo hunters (94%), with only two hunting events (6%) involving pairs of conspecifics simultaneously targeting the same vole. Both joint hunting events attempts involved two juveniles unsuccessfully chasing but not capturing the targeted vole (Table S2).

### Social aspects of vole consumption: competition and tolerance

Competitive interactions over voles were characterized by simple displacements of one squirrel by the aggressor or by the aggressor directly chasing the recipient of aggression in an attempt to steal the food item. Competitive interactions were mainly observed outside of hunting attempts, ensuing after only 5 of the successful hunting attempts. Additionally, we documented a total of 8 competitive interactions over vole carcasses that were already dead upon the start of our observations. Competitive interactions involved attempting to steal a vole carcass and were sometimes accompanied by agonistic behaviors; these included attempted theft, displacing, pushing, biting, or chasing (for more details, see Table S2). On one occasion, a producer of a vole carcass also vocalized at a conspecific apparently attempting to kleptoparasitize a vole. As with hunting and consumption, the age and sex of squirrels did not predict competitive interactions over voles (*p* = 0.62, Fig. 5c). On two occasions, consumption of voles also occurred within close proximity of socially tolerant conspecifics. On the first occasion, an adult male tolerated a juvenile female consuming the remainder of his hunted prey. On the second occasion, an adult female was consuming a freshly hunted vole in close proximity (< 1 m) to two newly emerged juveniles. Both juveniles were observed eating several pieces of the adult female’s vole (Table S2).

## Discussion

We provided the first evidence of California ground squirrels hunting, killing and consuming California voles in nature, offering new insights into the potential benefits of behavioral flexibility in foraging associated with a food pulse in the environment. Following an unusual increase in vole numbers (iNaturalist community 2024), we documented 74 observations of vole hunting and/or consumption across seven weeks for the first time in our population in the twelfth year of our long-term study. Interestingly, vole hunting and consumption were not concentrated in areas with a high density of vole burrows. Instead, these interactions were the greatest in areas of high ground squirrel burrow density, which aligns with our observations that voles did not only populate vole burrows but also used abandoned squirrel burrows. Moreover, consistent observations of vole hunting across the summer months throughout our study area, with a peak during the first two weeks of July, suggest that vole hunting behavior may be more widespread in California ground squirrels than previously known, and likely emerged, at least in part, in response to a temporary increase in availability of prey. Our observations of vole hunting extend previous reports of cannibalism of juvenile conspecifics and isolated predation events by squirrels on heterospecific adults (Sandberg and Banta 1973; Trulio 1996). The widespread nature of vole hunting in our population fundamentally changes our understanding of this primarily granivorous species, suggesting that they are considerably more flexible in their diet than previously assumed. Thus, our evidence extends earlier findings and further suggests that this species might be best characterized as an opportunistic omnivore, rather than a granivore.

Hunting is widespread among many social mammals, but strategies differ significantly among species. For example, carnivorous mammals living in fission–fusion societies may hunt prey on their own (singletons) or in small hunting parties of various sizes (e.g., African lions, spotted hyenas; Packer et al. 1990; Smith et al. 2008). Our observations of vole hunting suggest that hunting by California ground squirrels of California voles is largely a solo activity. We interpret the two isolated incidents of simultaneous hunting attempts on the same vole by two juvenile squirrels in our population as likely coincidental rather than as cooperative hunting attempts.

In many predators, hunting success is shaped by the age of the hunter (Brandt 1984; Holekamp et al. 1997; Funston et al. 2001; Smith and Holekamp 2023), especially when younger animals need to acquire or finetune their hunting skills either by trial and error or through social learning from adults (Kitowski 2009; Hilborn et al. 2012). Yet, we found no apparent age (or sex) bias in participation in vole hunting nor hunting success. This is somewhat surprising given that vole hunting requires skills and is not without risk, in particular for smaller (juvenile) animals. On several occasions, we observed voles fighting back when captured by a squirrel and inflicting bites on their capturer, sometimes resulting in squirrels releasing their prey.

In many carnivorous mammals, contest competition, involving aggression or dominance, ensues immediately after kills, particularly when food is limited or monopolizable (Frank 1986; Ramos-Fernández et al. 2006; Houle and Wrangham 2021). In other species, including humans, meat may be shared among group members (Smith et al. 2012; Creel and Creel 1991; Packer et al. 2001) or used to provision young (Holekamp and Smale 1990; Quinlan and Quinlan 2008; Geipel et al. 2013). California ground squirrels typically feed on non-monopolizable seeds or grasses (Smith et al. 2016), and direct competition over food resources is therefore rare. Yet here we documented squirrels repeatedly engaging in competition over depredated voles. This is perhaps because the energy contained in a single vole far outweighs that of more common food items, such as seeds or grasses. This may also explain why a ground squirrel vocalized at a conspecific during an agonistic encounter, a behavioral response that is usually only elicited by a threatening stimulus (e.g., a predator; Hanson and Coss 2001). While we have not observed instances of active meat-sharing in our squirrel population, multiple occasions of social tolerance during feeding have occurred during which squirrels picked up the vole remains hunted by other squirrels without agonistic interactions ensuing. An open question that remains is whether underlying factors such as kinship or familiarity predict competition over vole carcasses and/or social tolerance during feeding, as has been observed in other species (Smith et al. (2007); Smith et al. (2023); Griffiths and Armstrong 2002; Silk et al. 2013; Dale et al. 2017).

Another outstanding question is whether California ground squirrels are genetically predisposed to engage in hunting behavior when the opportunity presents itself or if hunting is socially learned (Boyd and Richerson 1996). The widespread nature of vole hunting across our study population and site, combined with previous reports of infanticide (Trulio et al. 1986; Trulio 1996), and hunting/killing of small prey in this species (see Table S1), seem to suggest the former. Studying the potential influence of social learning on the emergence, spread, and techniques underlying the direct hunting and consumption of adult vertebrate prey in ground squirrels is nonetheless an exciting new avenue for future research. More broadly, we contribute to growing evidence suggesting that hunting strategies and techniques may be less refined for opportunistic hunters compared to more carnivorous species of rodents (Langley 2021).

The patterns documented here might contribute to population and community dynamics in California grasslands. Access to an energetically rich and ephemeral food source, for example, may have positive fitness consequences for California ground squirrels, as seen in other rodent populations (Hoogland and Brown 2016). With respect to disease transmission, close associations between ground squirrels and voles could influence zoonoses. Although we observed no signs of disease in our study population, the behaviors here could influence host–parasite dynamics. California voles (Stark 2002) and ground squirrels (Smith et al. 2021) are endemic reservoirs for plague, both carrying fleas. Increased contact rates between these species could negatively influence rodent population sizes and, in turn, influence community processes. Future studies are therefore required to track the effects, if any, of innovative hunting behavior by the California ground squirrel on ecological processes at multiple levels of organization.

## Supplementary Information

Below is the link to the electronic supplementary material.

Supplementary file1 (JPG 471 KB)

Supplementary file2 (JPG 411 KB)

Supplementary file3 (JPG 706 KB)

Supplementary file4 (DOCX 116 KB)

Supplementary file5 (JPG 662 KB)

Supplementary file6 (JPG 843 KB)

Supplementary file7 (JPG 1230 KB)

Supplementary file8 (JPG 631 KB)

Supplementary file9 (JPG 434 KB)

Supplementary file10 (JPG 720 KB)

Supplementary file11 (JPG 364 KB)

Supplementary file12 (JPG 528 KB)

## Data Availability

All observational data are provided in the electronic supplementary material.
