# Supplementary figures and images for: Vole hunting: novel predatory and carnivorous behavior by California ground squirrels
Source: J Ethol. 2024 Dec 18;43(1):3–12. doi: 10.1007/s10164-024-00832-6 (PMC11717845; doi:10.1007/s10164-024-00832-6)

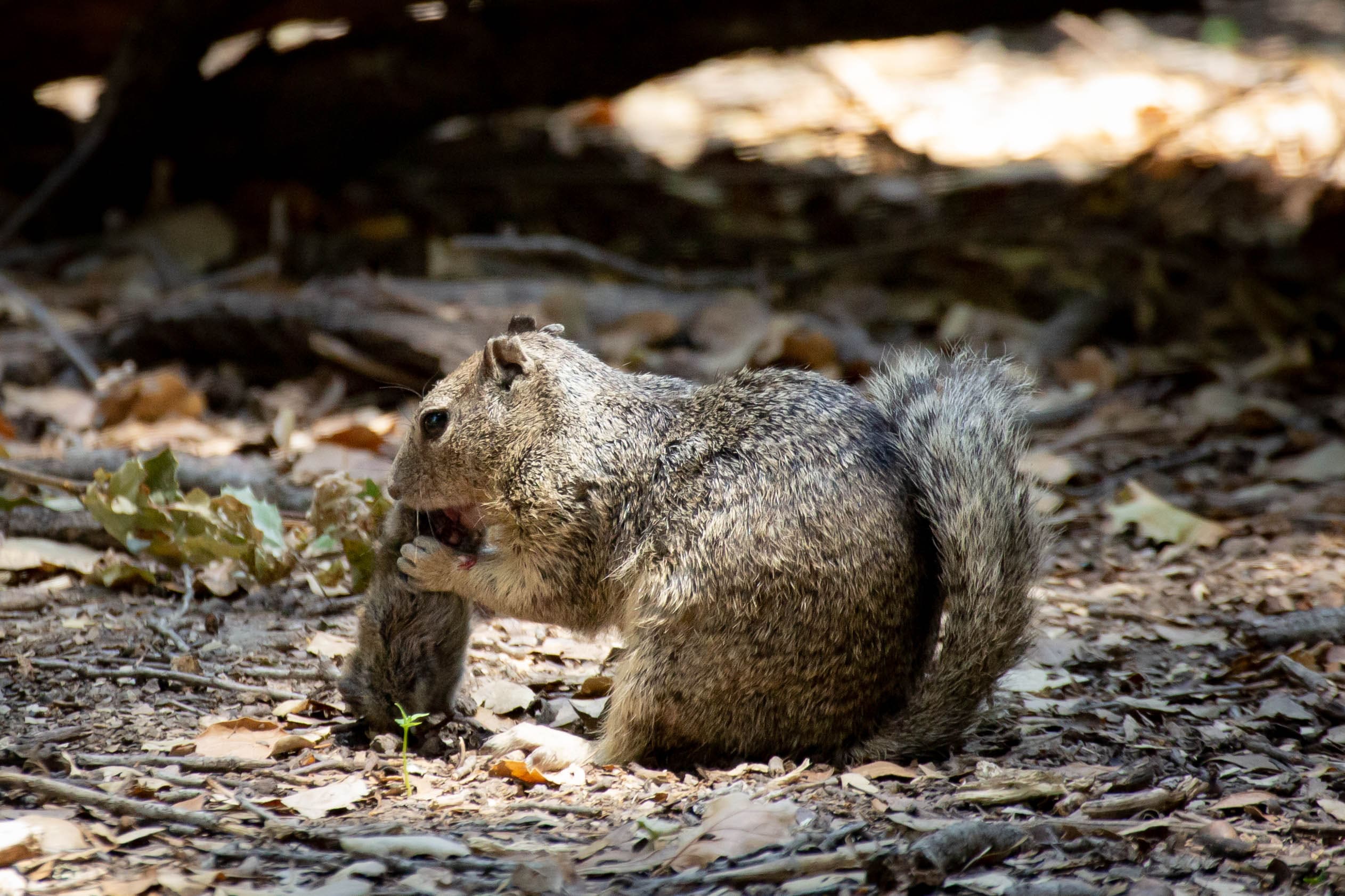

Supplement: Supplementary file 1 — Supplementary file1 (JPG 471 KB) [file 10164_2024_832_MOESM1_ESM.jpg]

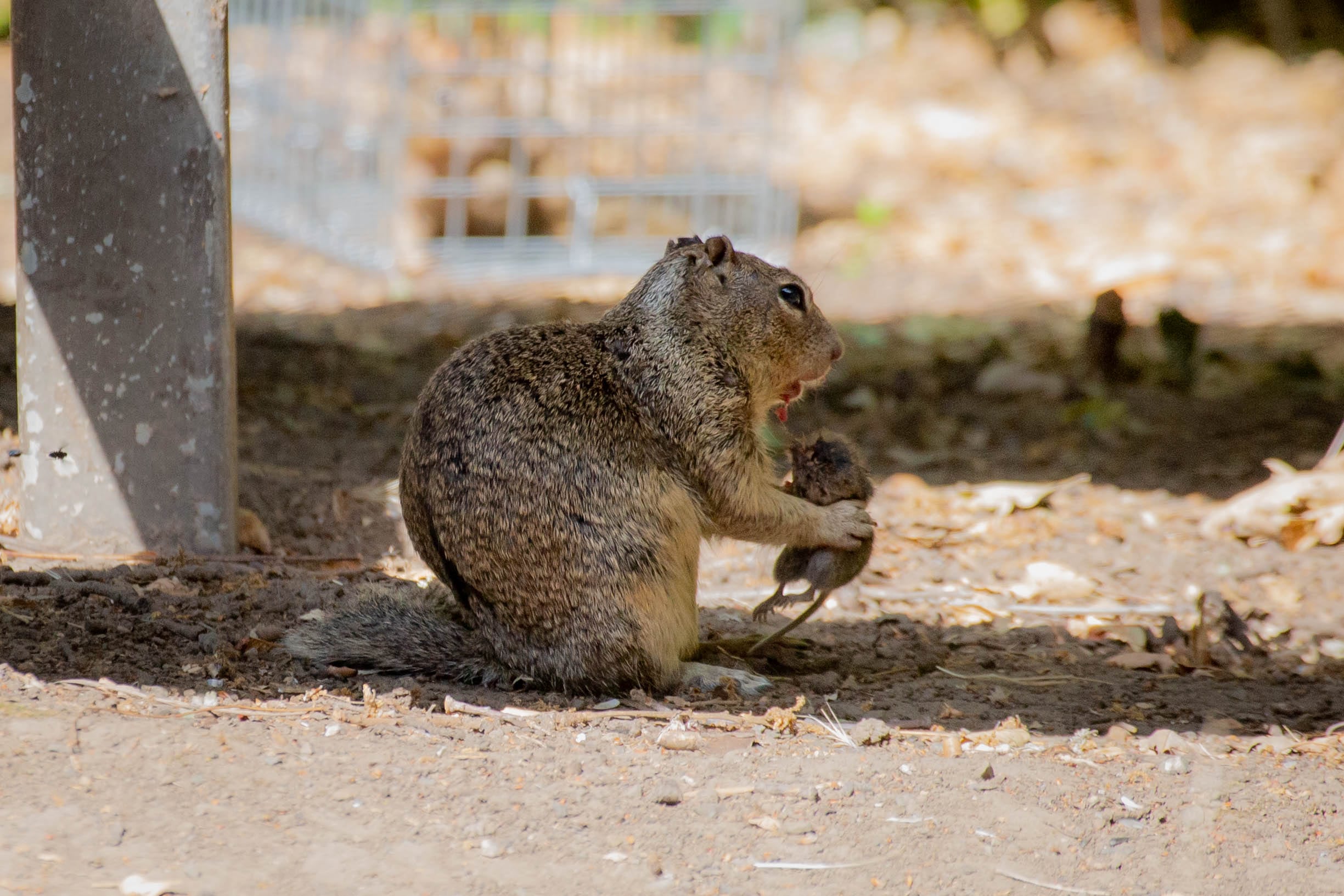

Supplement: Supplementary file 2 — Supplementary file2 (JPG 411 KB) [file 10164_2024_832_MOESM2_ESM.jpg]

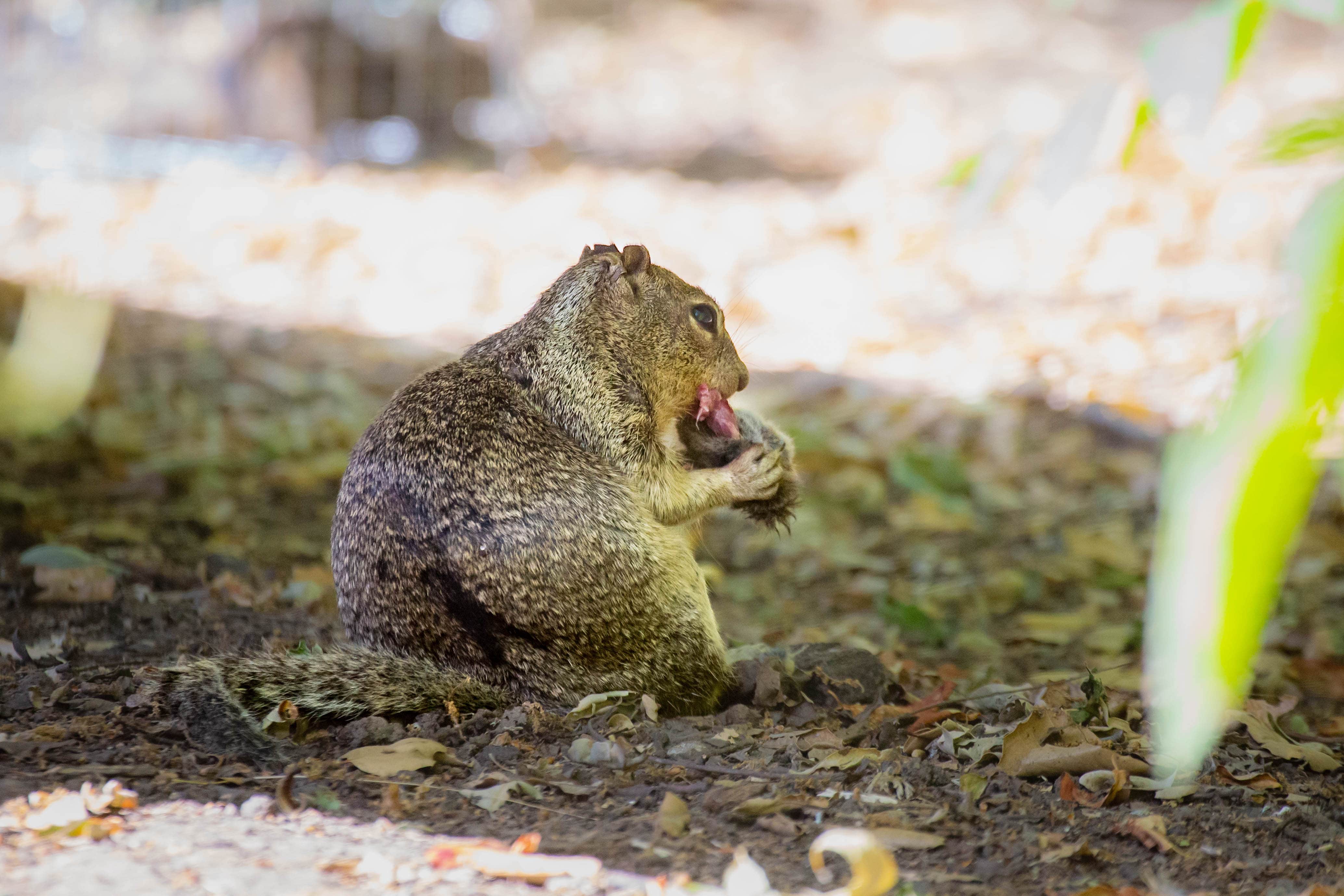

Supplement: Supplementary file 3 — Supplementary file3 (JPG 706 KB) [file 10164_2024_832_MOESM3_ESM.jpg]

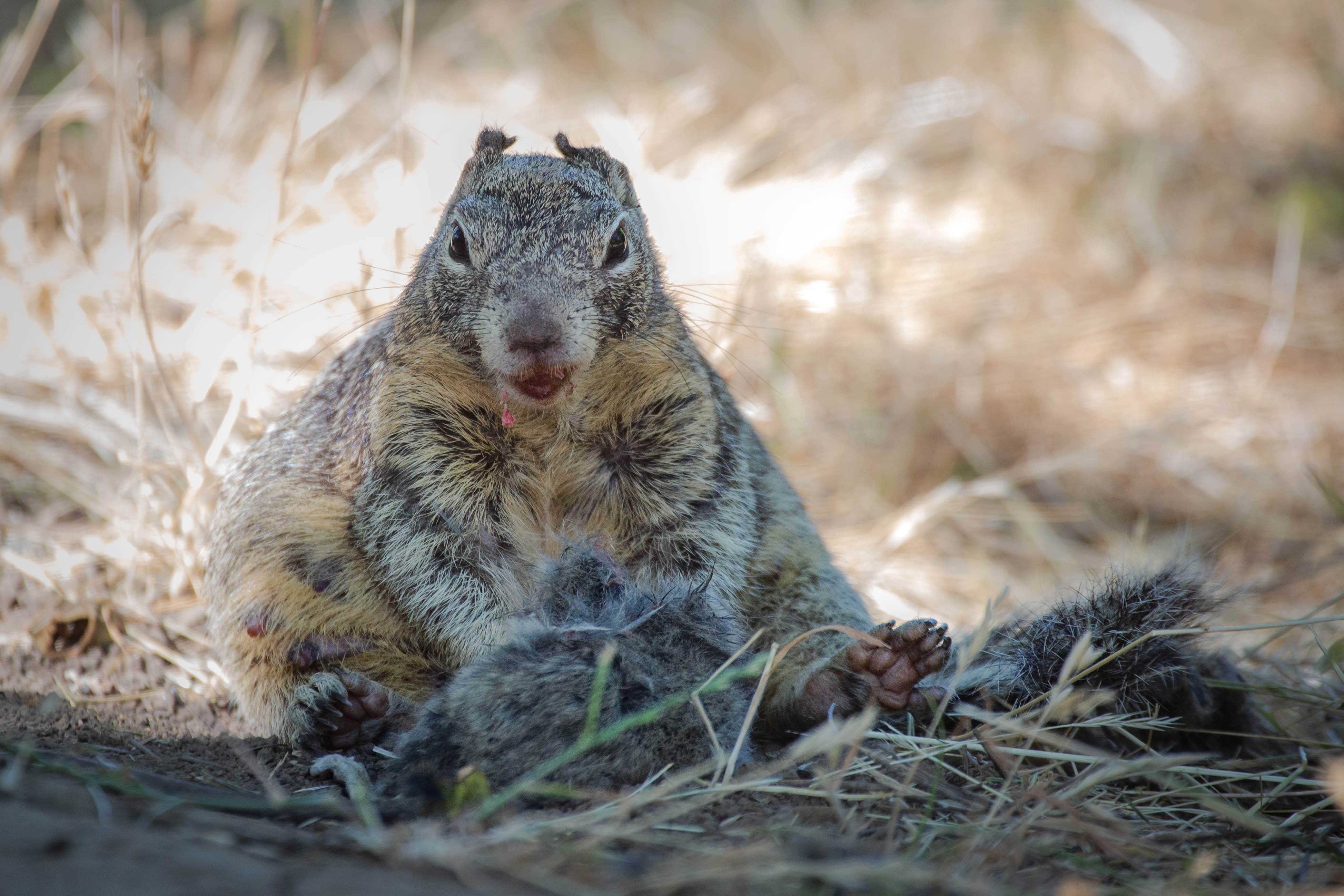

Supplement: Supplementary file 5 — Supplementary file5 (JPG 662 KB) [file 10164_2024_832_MOESM5_ESM.jpg]

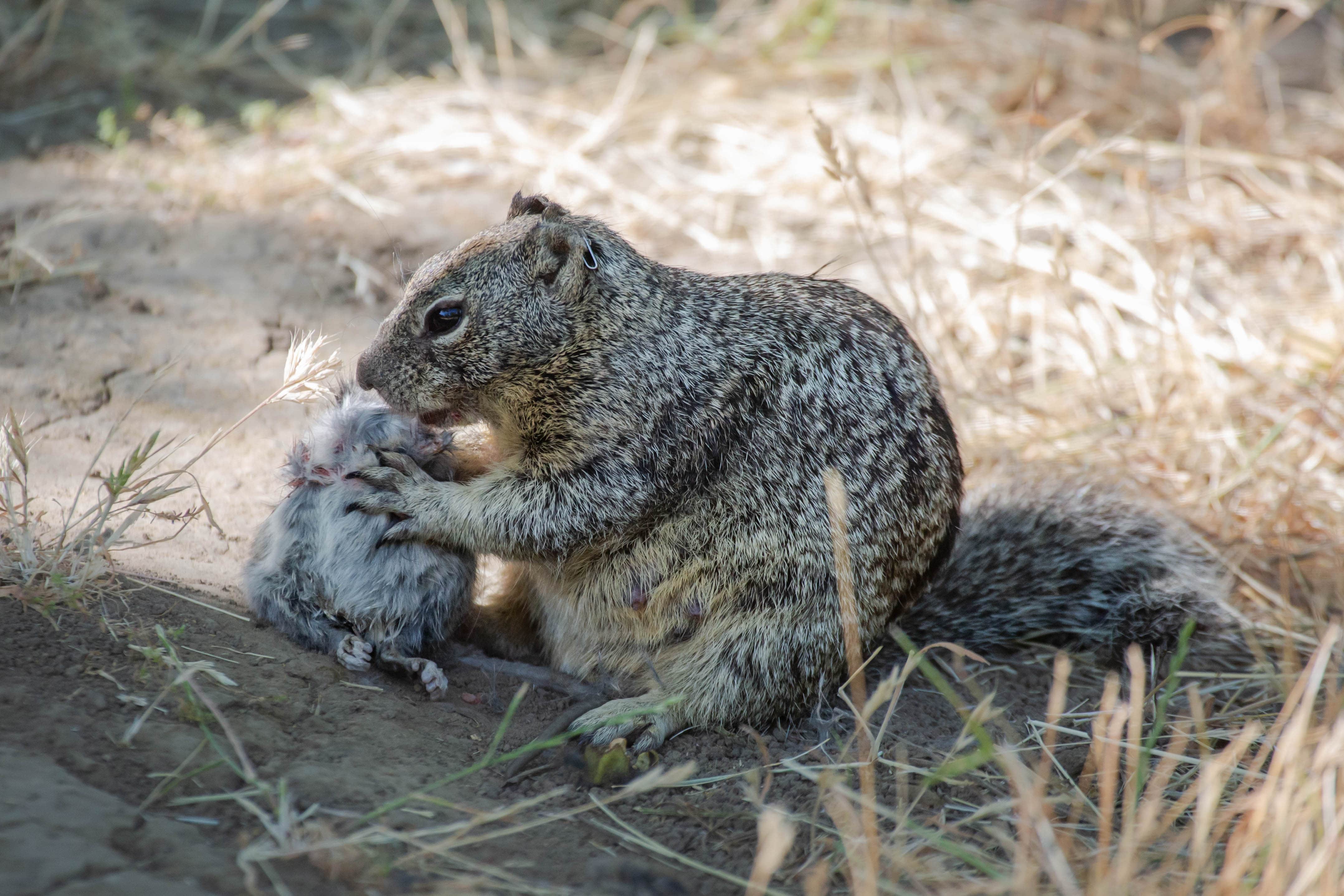

Supplement: Supplementary file 6 — Supplementary file6 (JPG 843 KB) [file 10164_2024_832_MOESM6_ESM.jpg]

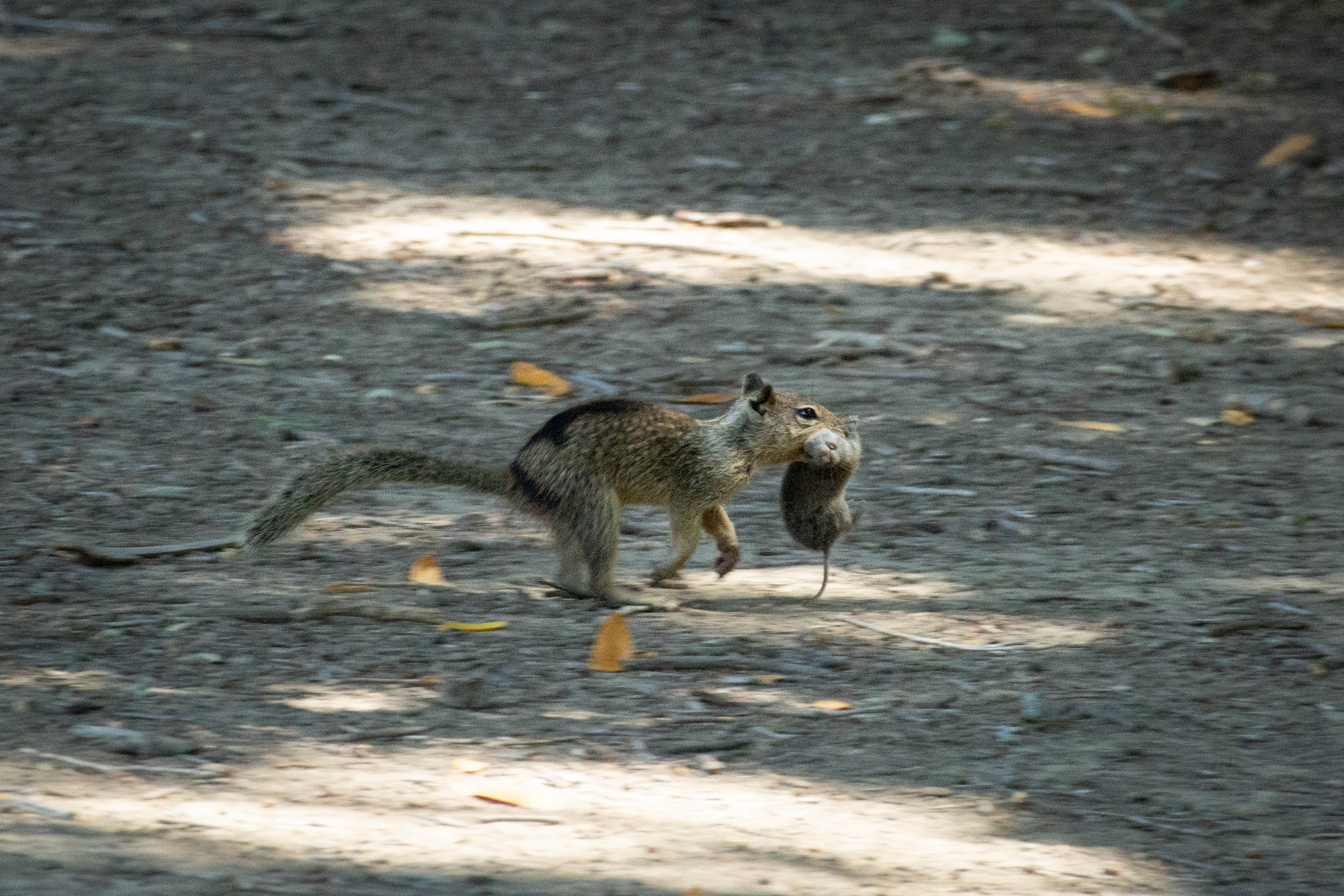

Supplement: Supplementary file 7 — Supplementary file7 (JPG 1230 KB) [file 10164_2024_832_MOESM7_ESM.jpg]

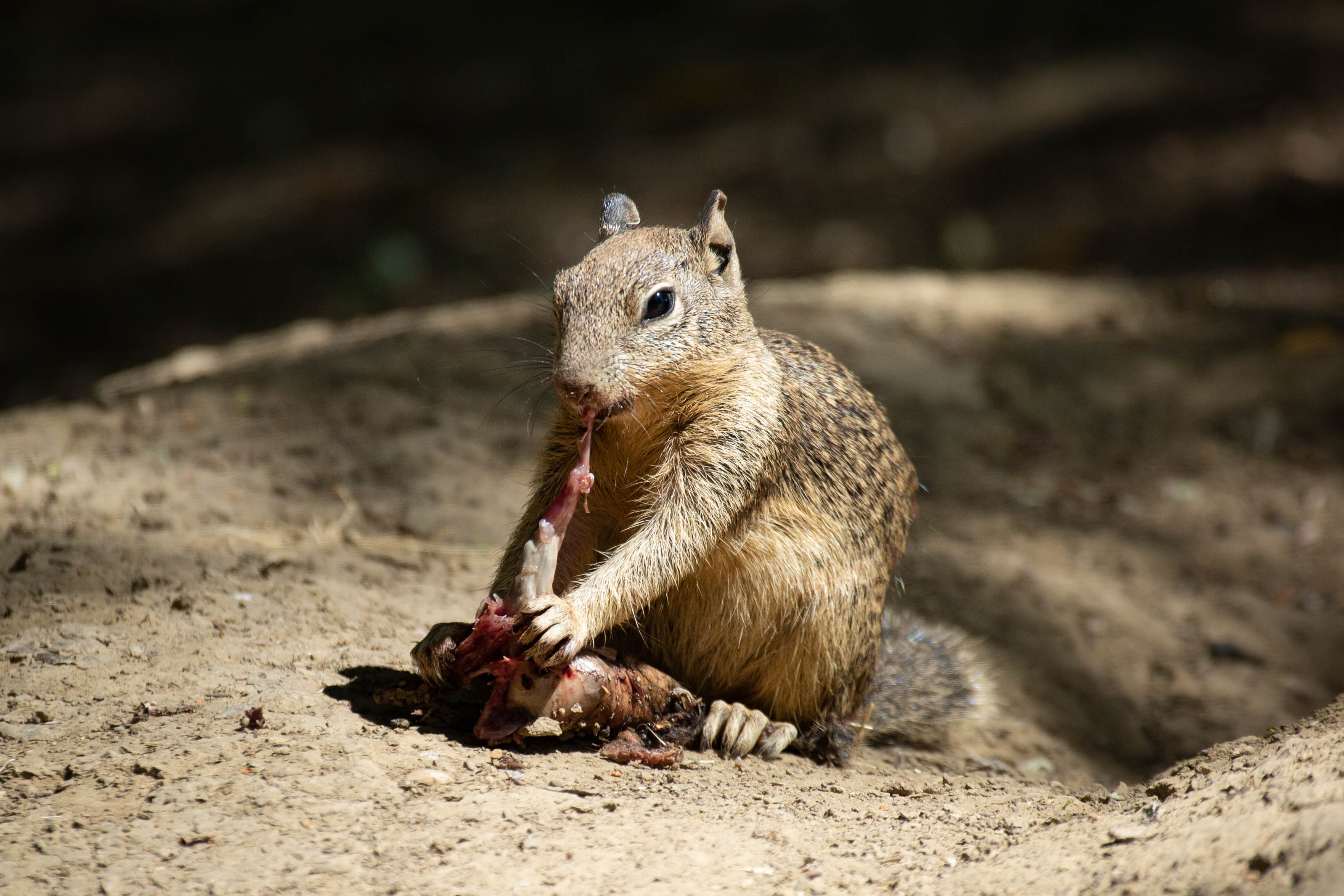

Supplement: Supplementary file 8 — Supplementary file8 (JPG 631 KB) [file 10164_2024_832_MOESM8_ESM.jpg]

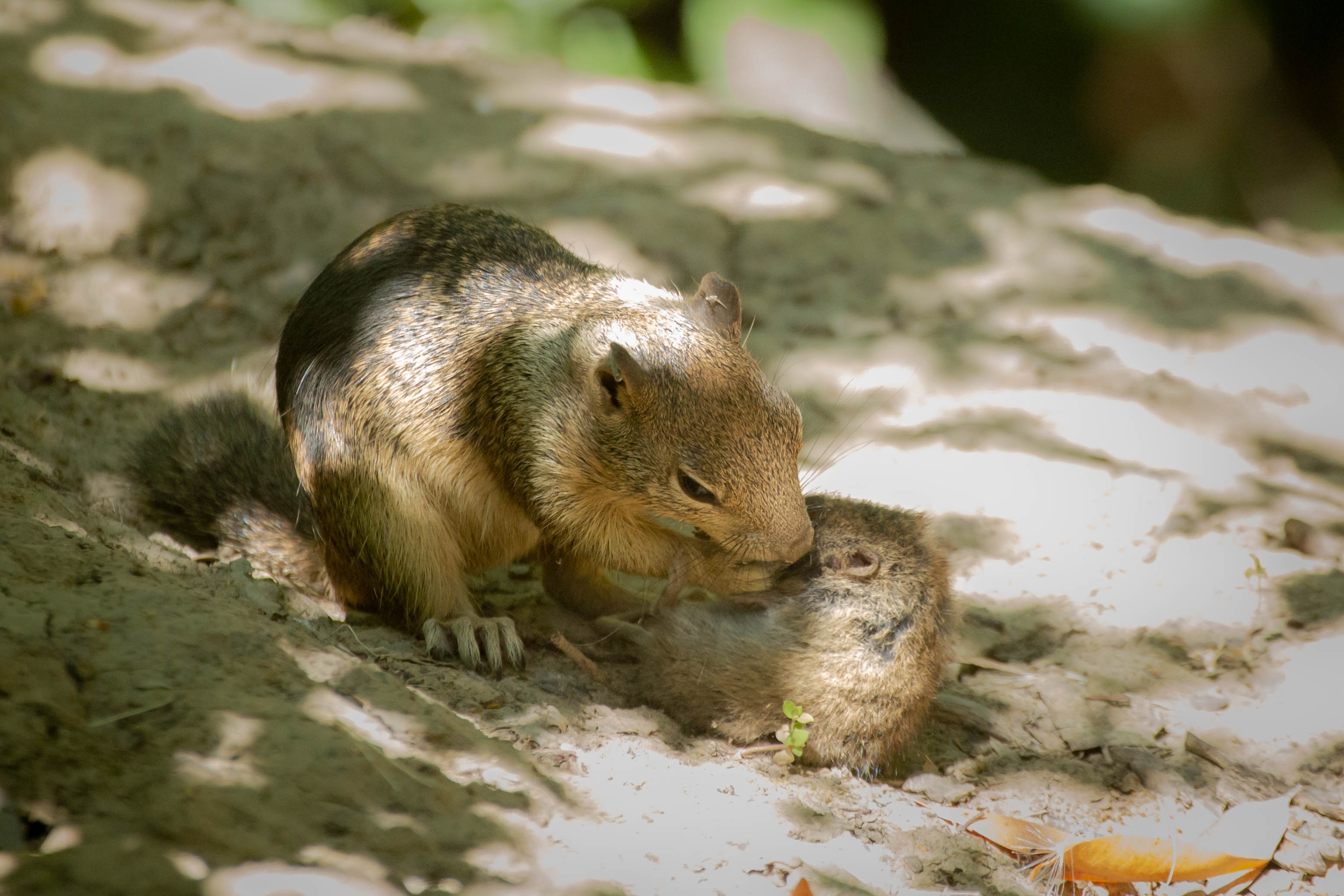

Supplement: Supplementary file 9 — Supplementary file9 (JPG 434 KB) [file 10164_2024_832_MOESM9_ESM.jpg]

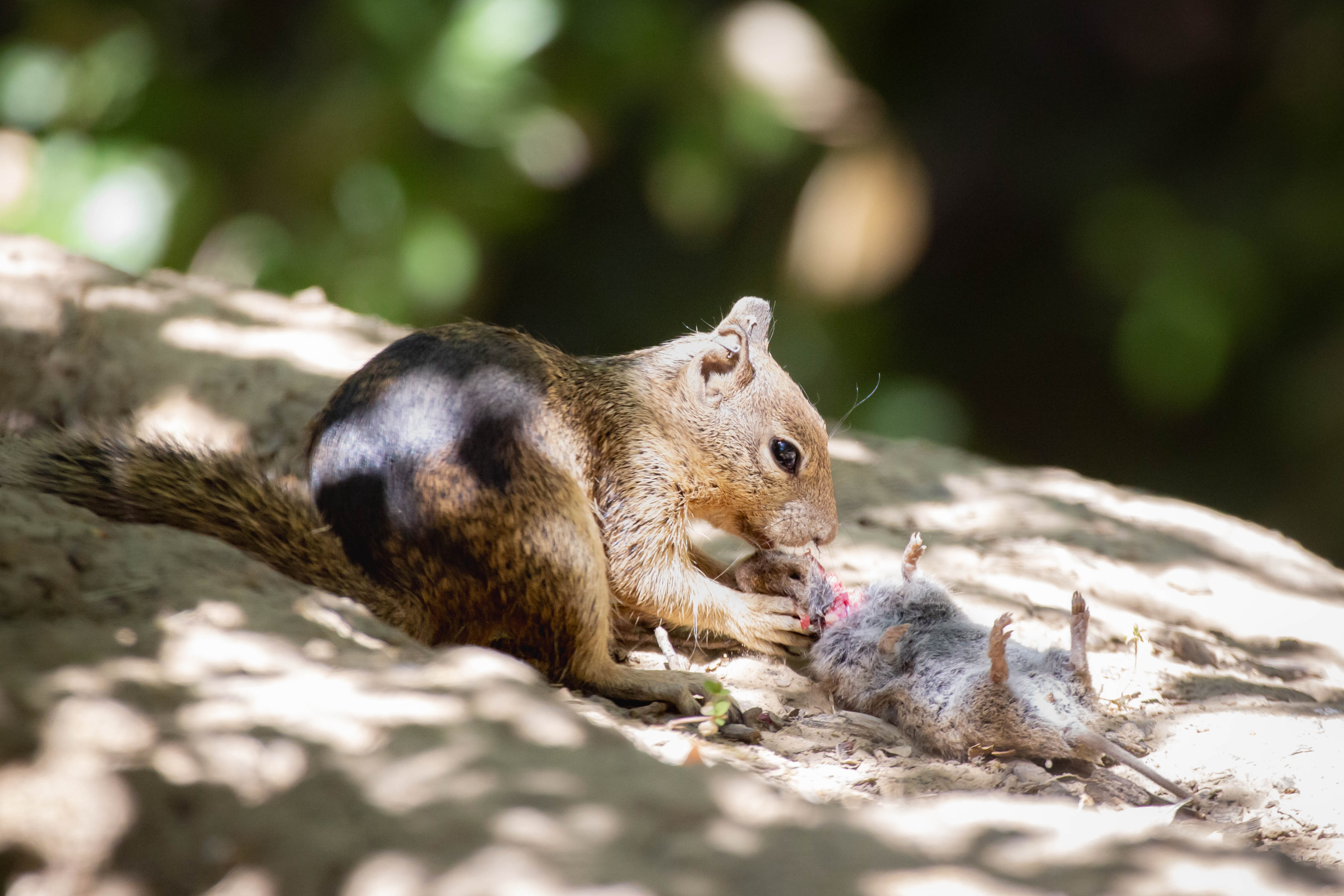

Supplement: Supplementary file 10 — Supplementary file10 (JPG 720 KB) [file 10164_2024_832_MOESM10_ESM.jpg]

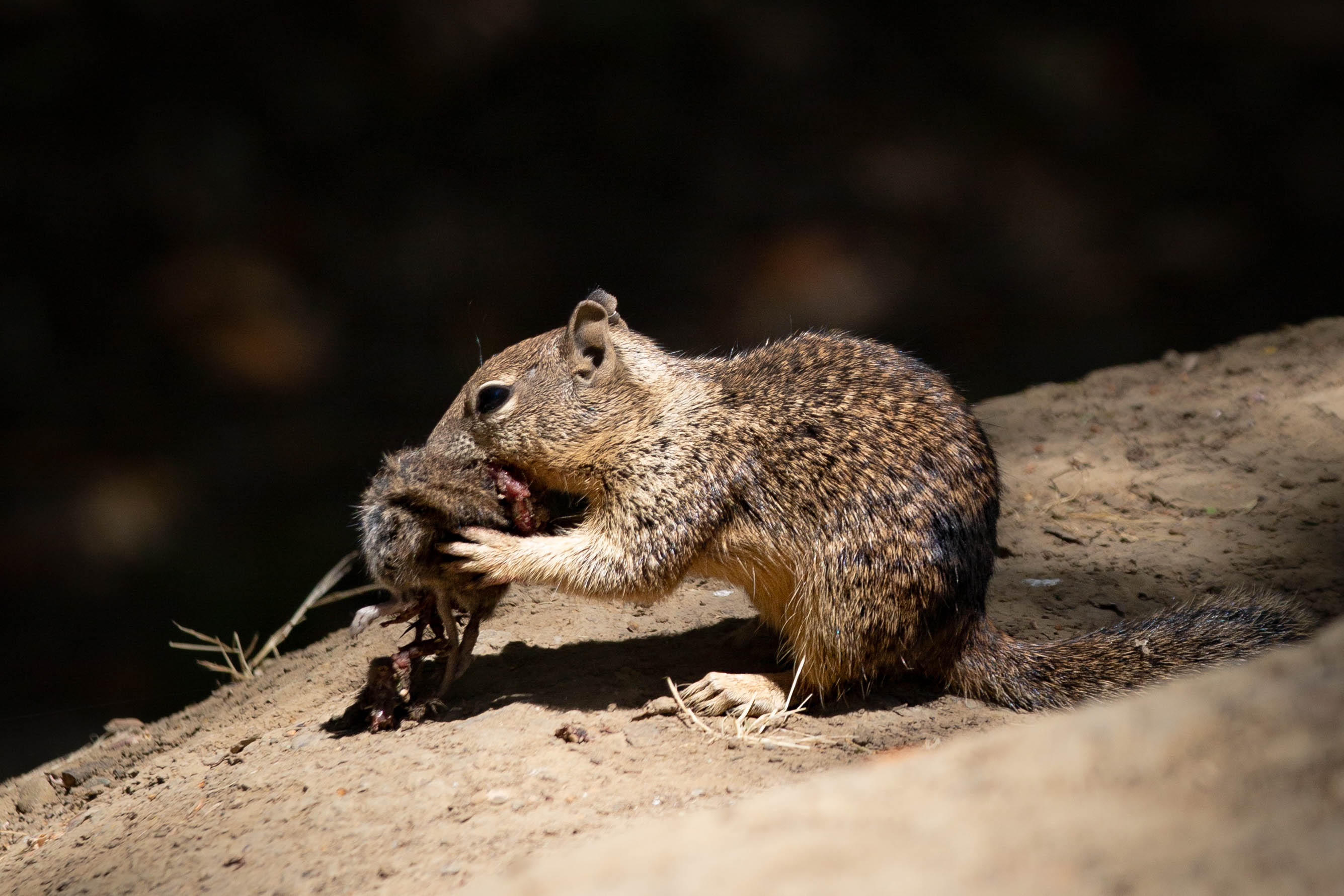

Supplement: Supplementary file 11 — Supplementary file11 (JPG 364 KB) [file 10164_2024_832_MOESM11_ESM.jpg]

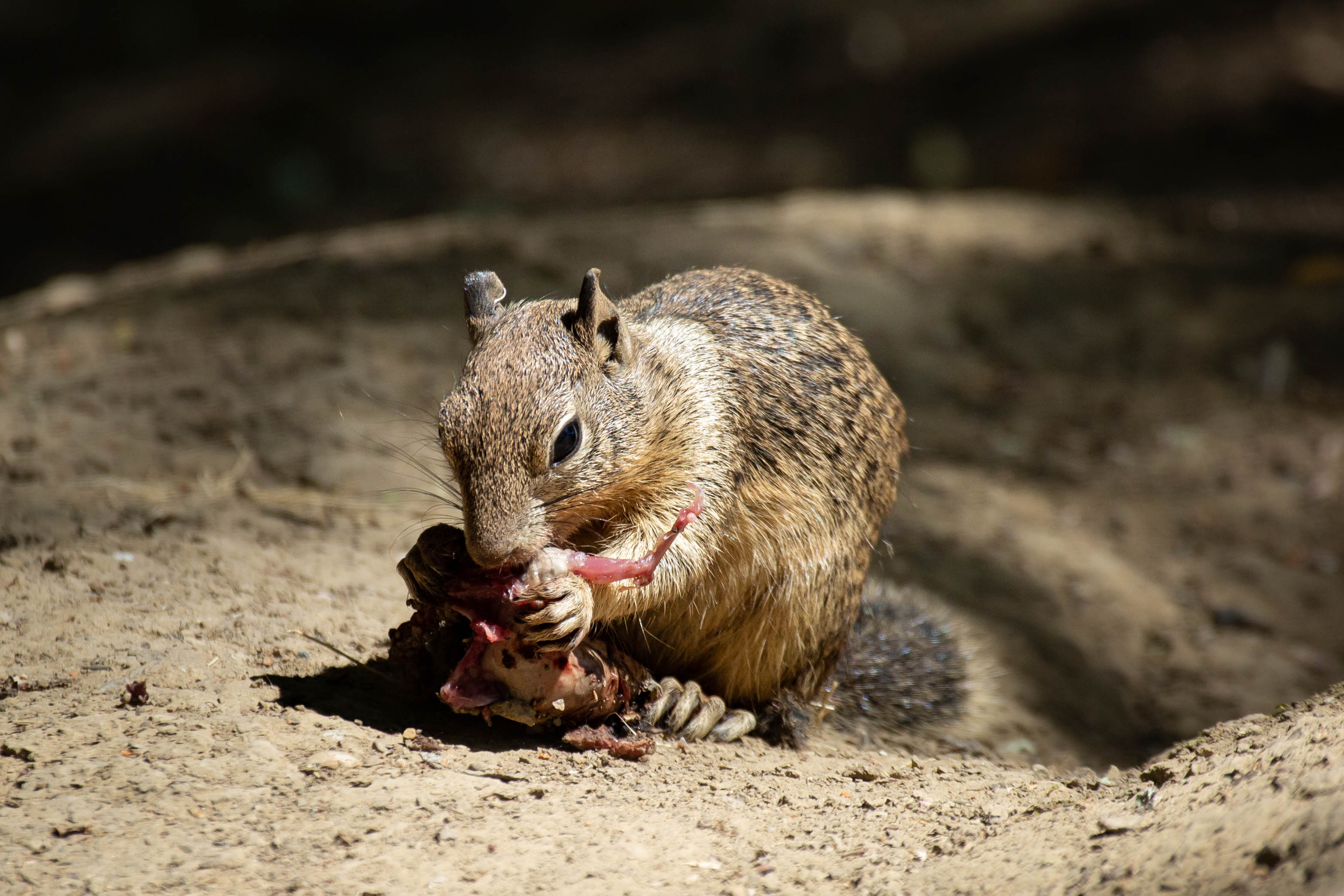

Supplement: Supplementary file 12 — Supplementary file12 (JPG 528 KB) [file 10164_2024_832_MOESM12_ESM.jpg]
