# Supplementary material for: Vole hunting: novel predatory and carnivorous behavior by California ground squirrels
Source: J Ethol. 2024 Dec 18;43(1):3–12. doi: 10.1007/s10164-024-00832-6 (PMC11717845; doi:10.1007/s10164-024-00832-6)
Supplement: Supplementary file 4 — Supplementary file4 (DOCX 116 KB) [file 10164_2024_832_MOESM4_ESM.docx]

**Supplementary material: Vole hunting: First video and photographic evidence of novel predatory and carnivorous behavior by California ground squirrels**

**Jennifer E. Smith^1^, Joey E. Ingbretson^1^, Mackenzie M. Miner^1^, Ella C. Oestreicher^1^, Mari L. Podas^1^, Tia A. Ravara^1^, Lupin M. L. Teles^2,3^, Jada C. Wahl^1^, Lucy M. Todd^2^, Sonja Wild^2^**

^1^Department of Biology, University of Wisconsin – Eau Claire, Wisconsin 54701, USA

^2^Department of Environmental Science and Policy, University of California, Davis, California 95616, USA

^3^Institute of Biological Sciences and Health, Federal University of Alagoas, Maceió, Alagoas 57072-900, Brazil

Corresponding author: [swild@ucdavis.edu](mailto:swild@ucdavis.edu)

**Table S1: Evidence for killing of heterospecifics among Sciuridae in the literature**

| **Species** | **Direct killing (hunting) or consumption - evidence** |
| --- | --- |
| **White-tailed antelope squirrel**  (*Ammospermophilus leucurus*) | Direct observation of killing and consumption of a pocket mouse (Morgart 1985) |
| **Round-tailed ground squirrel**  (*Xerospermophilus tereticaudus*) | Direct observation of stalking, killing, and eating English sparrows (Bradley 1968) |
| **White-tailed prairie dog** (*Cynomys leucurus*) | Killed (not consumed) Wyoming ground squirrel (*Urocitellus elegans*) (Hoogland and Brown 2016) |
| **Mexican ground squirrel**  (*Ictidomys mexicanus*) | Observed ground squirrel holding a small, live cottontail rabbit (*Sylvilagus* spp.) by the neck, but human interrupted this predation attempt by shooting ground squirrel (Packard 1958) |
| **13-lined ground squirrel** (*I. tridecemlineatus*) | Direct killing of young domestic chicken (Bailey 1923); NOTE: Juvenile rather than adult prey. |
| **Yellow-bellied marmot** (*Marmota flaviventris*) | Direct observation of one direct kill; two instances of consumption only (Armitage et al. 1979) |
| **Alpine marmot** (*Marmota marmota*) | Opportunistic predation on newborn Alpine ibex (*Capra ibex*) in zoo (Böhm and Landmann 2021) |
| **California ground squirrel**  (*Otospermophilus beecheyi*) | Direct killing of 'fairly large chicken'; failed to kill young quail but consumed quail eggs (Sumner and Dixon 1953); Direct killing of moles (*Scapanus latimanus*) and lizards (Trulio et al. 1986) |
| **European ground squirrel**  (*Spermophilus citellus*) | Direct observations of active killing and consumption of a young Eurasian tree sparrow (Kachamakova et al. 2022). NOTE: Juvenile rather than adult prey. |
| **Franklin's ground squirrel**  *(Poliocitellus franklinii)* | Direct observation of the killing and consumption of a rabbit (Johnson 1922); States “it is known” to eat rabbit, wild mice, and capture chicken (Howell 1938) |
| **Eastern gray squirrel**  (*Sciurus carolinensis*) | Direct observation of the killing of a chicken (Bailey 1923); hunting and consuming an unknown species of live fish (Sutton et al. 2020) |
| **Fox squirrel** (*S. niger***)** | Direct observation of predation of a juvenile blue jay (*Cyanocitta cristata*) (Shaffer and Baker 1991) |
| **Red squirrel** (*S. vulgaris*) | Attempted consumption of three poultry chickens (Hatt 1929); direct observation of a killing of a house sparrow (Nero 1987) |
| **Eastern chipmunk** (*Tamias striatus*) | Direct observation of the killing of a live frog (Hesterberg 1950) and the killing (but not consuming of) a maritime garter snake, *Thamnophis sirtalis pallidulus* (Abolit and Gilhen 2011) |
| **Arctic ground squirrel** (*Urocitellus parryii*) | Directly killed and (partially or completely) consumed lemmings (Boonstra et al. 2011) |
| **Rock squirrel** (*Otospermophilus variegatus*) | Caught and ate young wild turkeys (Cook and Henry 1940) |

**References**

Abolit D, Gilhen J (2011) Eastern chipmunk, *Tamias striatus* attack on a maritime garter snake. Can Field Nat 125:55–57

Armitage KB, Johns D, Andersen DC (1979) Cannibalism among yellow-bellied marmots. Source: Journal of Mammalogy 60:205–207

Bailey B (1923) Meat-bating propensities of some rodents of Minnesota. J Mammal 4:129–129. https://doi.org/10.1093/JMAMMAL/4.2.129

Böhm C, Landmann A (2021) Carnivory in the alpine marmot (*Marmota marmota*): An underestimated phenomenon? Ethol Ecol Evol 33:184–190. https://doi.org/10.1080/03949370.2020.1837964

Boonstra R, Krebs CJ, Kanter M (2011) Arctic ground squirrel predation on collared lemmings. Can J Zool 68:757–760. https://doi.org/10.1139/Z90-109

Bradley WG (1968) Food habits of the antelope ground squirrel in southern Nevada. J Mammal 49:14–21

Cook AH, Henry WH (1940) Texas rock squirrels catch and eat young wild turkeys. J Mammal 21:92

Hatt RT (1929) The red squirrel: Its life history and food habits. Roosevelt Wild Life Forest Experiment Station 2:10–146

Hesterberg GA (1950) Chipmunk eats frog. J Mammal 31:350--35l

Hoogland JL, Brown CR (2016) Prairie dogs increase fitness by killing interspecific competitors. Proceedings of the Royal Society B 283:20160144. https://doi.org/10.1098/rspb.2016.0144

Howell AH (1938) North American Fauna. U.S. Government Printing Office

Johnson AM (1922) An observation on the carnivorous propensities of the gray gopher. J Mammal 3:187–187. https://doi.org/10.1093/JMAMMAL/3.3.187

Kachamakova M, Koynova T, Tsvetkov R, Koshev Y (2022) First evidence for active carnivorous predation in the European ground squirrel. Acta Ethol 25:191–193. https://doi.org/10.1007/S10211-022-00399-W/FIGURES/1

Morgart JR (1985) Carnivorous behavior by a white-tailed antelope ground squirrel, *Ammospermophilus leucurus*. Southwest Nat 30:304–305

Nero RW (1987) House sparrow killed by red squirrel. Blue Jay 45:180–181. https://doi.org/10.29173/bluejay5023

Packard RL (1958) Carnivorous behavior in the Mexican ground squirrel. J Mammal 39:154

Shaffer B, Baker B (1991) Observations of predation on a juvenile blue jay *Cyanocitta cristata* by a fox squirrel, *Sciurus niger*. Texas Journal of Science 43:105–106

Sumner L, Dixon JS (1953) Birds and Mammals of the Sierra Nevada: With Records from Sequoia and Kings. University of California Press, Berkeley, CA

Sutton AO, Fuirst M, Bill K, et al (2020) Into the drink: Observation of a novel hunting technique employed by an eastern gray squirrel (*Sciurus carolinensis*). The Canadian Field Naturalist 134:42–44. https://doi.org/10.22621/cfn.v134

Trulio LA, Loughry WJ, Hennessy DF, Owings DH (1986) Infanticide in California ground squirrels. Anim Behav 34:291–294. https://doi.org/10.1016/0003-3472(86)90037-0

**Table S2: Observation of hunting of, consumption of, or competition over California voles by California ground squirrels in summer 2024**

| **#** | **Obs. day** | **Date** | **Fur mark** | **Sex** | **Age** | **Description** | **Activity** | **Successful hunt?** |
| --- | --- | --- | --- | --- | --- | --- | --- | --- |
| 1 | no | 10-Jun-24 | merge_sign | M | A | merge_sign feeding on a vole for several minutes near trapping station, holding it vertically between front paws. Consumed the head first. | consumption | NA |
| 2 | yes | 13-Jun-24 | AG  Aries | F  F | A  A | AG running to Tree L with a dead vole in her mouth. Sitting in front of burrow entrance holding it vertically between her front paws and eating from the head down. After about 10 minutes, Aries approached (presumably in an attempt to steal the vole), but AG alarm called in her direction and then aggressively chased her away towards Tree H, before returning to Tree L and continuing to consume the vole. | competition, consumption | NA |
| 3 | yes | 13-Jun-24 | Aries | F | A | Aries stalks, then captures live vole, picking it up with her mouth three times and dropping in between as the vole fights back. On the third attempt, she flinches and drops the vole (likely as a reaction to getting bitten), and briefly rubs her chin on the dirt before moving away. The vole is visibly injured and limps away only after several minutes of immobility. | hunting | no |
| 4 | yes | 14-Jun-24 | Cyclops  AG  snakehead | F  F  F | P  A  A | Cyclops appearing from a burrow with a dead vole. AG approaches and attempts to steal the vole, but intervention by snakehead (who is presumably mother of cyclops), chases AG away. AG returns to pick up dropped vole | consumption, competition | NA |
| 5 | no | 15-Jun-24 | merge_sign | M | A | Merge_sign feeding on a vole near tree Z, moved over to sunnyside on approach and continued to consume the vole under a picnic table before disappearing into the bushes. | consumption | NA |
| 6 | yes | 27-Jun-24 | Insect  half_note | F  F | P  P | Insect seen carrying a vole towards pine (start point unclear), it started feeding on the vole under the picnic table holding it vertically. After a few minutes, half_note approached in an attempt to steal the vole, displacing insect who disappeared into the bush still carrying the vole. A few minutes later, insect reappeared at oak with the partially eaten vole (only the bottom half left now), continuing to feed on it near a burrow entrance for about 10-15 minutes before discarding of the vole leftovers and starting to rest. | consumption, competition | NA |
| 7 | yes | 28-Jun-24 | Capital_K  backwards_C  UnID juvenile | M  M | P  A | Capital K stole a dead vole from a smaller unmarked juvenile and attempted to cache it unsuccessfully, moved off with it about a metre, cached it. About 15 min later backwards_C found it and started eating it for about 10 min, then took it into the woods out of sight. | competition, consumption, caching | NA |
| **#** | **Obs. day** | **Date** | **Fur mark** | **Sex** | **Age** | **Description** | **Activity** | **Successful hunt?** |
| 8 | yes | 28-Jun-24 | Cyclops | M | P | Cyclops notices a vole about 0.5 m from her, cyclops attacks the vole, they wrestle (the vole is fighting back), the vole escapes and runs into a squirrel burrow, cyclops follows, then emerges about a minute later without the vole. | hunting | no |
| 9 | yes | 28-Jun-24 | bucket | F | A | Bucket was seen running from tree E with a moving vole. Ran to D area and began consuming the vole. It first consumed the head, then the intestines over the span of 15 minutes. When only the tail and legs were left, bucket took it into the burrow. | hunting, consumption | yes |
| 10 | yes | 28-Jun-24 | barnacle | M | A | Small bones and scraps from bucket's vole were left at area D and barnacle was seen eating meat off of the leftover bones. | consumption | NA |
| 11 | yes | 01-Jul-24 | snakehead | F | A | Snakehead observed consuming vole upon arrival. No hunting observed. Consumption lasted over half an hour. | consumption | NA |
| 12 | yes | 01-Jul-24 | Bowtie  unID juvenile | M | A | 8:24 consuming vole at cedar, keeping it on the ground rather than holding it between front paws; 8:52 seen consuming (presumably the same) vole at pine. 8:58 an unID juvenile consuming remains from Bowtie's vole. | consumption | NA |
| 13 | yes | 01-Jul-24 | Cyclops | M | P | Seen carrying a vole to W Tree, and consuming it in front of a burrow entrance | consumption | NA |
| 14 | yes | 01-Jul-24 | seventy_six | F | A | Seen carrying a dead vole from N_tree to log_3 where it disappeared into the woods. No hunting observed. | consumption | NA |
| 15 | yes | 01-Jul-24 | Battery  bucket | F  F | A  A | Battery caught a live vole at tree J. Bucket chased battery, who took the vole over to tree E to eat it. The vole was still moving slightly when battery sat down at E. She tore out the neck and ate the head first. In response to squirrel alarm calls caused by two off-leash dogs, battery ran into a burrow in D area with the vole in her mouth. She reemerged from the burrow at 9:22 with no vole. | hunting, competition, consumption | yes |
| 16 | yes | 01-Jul-24 | UnID adult |  | A | Observed carrying a dead vole from M_tree to R_tree before disappearing into the woods. | consumption | NA |
| 17 | yes | 01-Jul-24 | seventy_six | F | A | Seventy_six attacked and killed vole at L_tree, then carried it to log_3 where it dug up dirt, presumably in an attempt to cache it. She then consumed part of the carcass, before carrying the vole into the woods. | hunting, consumption | yes |
| 18 | yes | 01-Jul-24 | backwards_C | M | A | Consuming vole at cedar | consumption | NA |
| **#** | **Obs. day** | **Date** | **Fur mark** | **Sex** | **Age** | **Description** | **Activity** | **Successful hunt?** |
| 19 | yes | 01-Jul-24 | UnID juvenile |  | P | Squirrel with swollen check seen consuming vole under bench | consumption | NA |
| 20 | yes | 01-Jul-24 | UnID juvenile |  | P | Squirrel with swollen check consuming dead vole, potentially the same vole as earlier | consumption | NA |
| 21 | yes | 01-Jul-24 | UnID adult |  | A | Squirrel carrying vole | consumption | NA |
| 22 | yes | 01-Jul-24 | UnID juvenile |  | P | Eating dead vole. Squirrel left for 2 minutes then returned to continue eating the head and internal organs of the vole. Left the back legs of vole when done. | consumption | NA |
| 23 | yes | 01-Jul-24 | UnID adult |  | A | Squirrel attacked vole, unsuccessfully. | hunting | no |
| 24 | yes | 01-Jul-24 | UnID adult |  | A | Unidentified adult captured a live vole and began consuming it. | hunting, consumption | yes |
| 25 | yes | 01-Jul-24 | UnID juvenile |  | P | Juvenile consuming dead vole, another small unmarked juvenile (probable littermate) sniffs it but walks away. | consumption | NA |
| 26 | yes | 07-Jul-24 | opera | F | P | Opera observed consuming a vole, no hunting observed. | consumption | NA |
| 27 | yes | 07-Jul-24 | UnID adult |  | A | Seen consuming a vole, killing was not observed. | consumption | NA |
| 28 | yes | 07-Jul-24 | Capital_K | M | P | Seen with live vole in mouth, before it stopped moving. Capital K then started consuming the vole. | hunting, consumption | yes |
| 29 | yes | 07-Jul-24 | Aries | F | A | Aries attacked and killed a vole at tree H. When she had eaten about half of the vole, she went into a burrow with the vole disturbed by a park visitor at the gate. She emerged from the burrow at 9:24 with no vole. | hunting, consumption | yes |
| 30 | yes | 07-Jul-24 | UnID adult |  | A | Consuming a dead vole, killing was not observed. | consumption | NA |
| 31 | yes | 07-Jul-24 | UnID juvenile |  | P | An unmarked juvenile chased and attacked a vole between tree M and N. They carried it to the road near pine and began consuming the vole. At 9:49 they went into the woods carrying the dead vole in their mouth. | hunting, consumption | yes |
| 32 | yes | 07-Jul-24 | UnID adult |  | A | Consuming a dead vole, killing was not observed. | consumption | NA |
| **#** | **Obs. day** | **Date** | **Fur mark** | **Sex** | **Age** | **Description** | **Activity** | **Successful hunt?** |
| 33 | no | 08-Jul-24 | Capital_K | M | P | Consuming dead vole | consumption | NA |
| 34 | no | 08-Jul-24 | Bowtie  Aries | M  F | A  A | Bowtie: 8:45 at Tree E: Chased, wrestled, and captured a vole. Went out of sight with the vole and came back 5min later with the dead vole in his mouth, then 8:47 at Tree H Aries heads over to Bowtie who is eating vole, but Bowtie stays with vole. | hunting, consumption, competition | yes |
| 35 | no | 08-Jul-24 | Bowtie  unID juvenile | M | A  P | bowtie stole dead vole from unID juvenile and consumed it. | consumption, competition | NA |
| 36 | no | 08-Jul-24 | Libra  capital_Z | M  F | P  P | Libra kills vole at Tree N, carries vole, and eats it at Tree F. At 9:13 at Tree F, Capital_Z chases Libra with the vole, unsuccessfully | hunting, consumption, competition | yes |
| 37 | no | 08-Jul-24 | Bowtie  unID juvenile  barnacle  Amazon | M    M  F | A  P  A  A | 9:22 at Tree C Bowtie unsuccessfully chases after vole. At 9:40 at Tree E bowtie, chases, pounces on and kills vole, and starts consuming it. At 10:13 at Tree E unID juvenile eats leftovers of vole from bowtie's hunt, followed by barnacle who eats some at 11:14. At 11:31 Amazon sniffs remains of dead vole | hunting, consumption | yes |
| 38 | yes | 10-Jul-24 | capital_K | M | P | Unsuccessfully chasing a vole who disappears into burrow (twice) | hunting | no |
| 39 | yes | 10-Jul-24 | UnID adult |  | A | Chases a vole back into its hole | hunting | no |
| 40 | yes | 10-Jul-24 | half_note | F | P | eating dead vole remains | consumption | NA |
| 41 | yes | 10-Jul-24 | bowtie | M | A | Unsuccessful vole chase | hunting | no |
| 42 | yes | 10-Jul-24 | Cyclops | M | P | Chase a vole back into its hole | hunting | no |
| 43 | yes | 10-Jul-24 | UnID adult |  | A | Unidentified adult carrying dead vole at tree E. | carry vole, consumption | NA |
| 44 | yes | 10-Jul-24 | Snakehead  unID juvenile | F | A  P | A vole popped out of a burrow in front of where snakehead was foraging. Snakehead immediately chased, wrestled and caught it, and then bit it on the neck to kill it. Snakehead then carried the vole to Tree W, took off the head, and began eating the insides; 9:53: @Tree W: seen a juvenile eating vole, suspected to be vole caught by snakehead | hunting, consumption | yes |
| **#** | **Obs. day** | **Date** | **Fur mark** | **Sex** | **Age** | **Description** | **Activity** | **Successful hunt?** |
| 45 | yes | 10-Jul-24 | bowtie | M | A | Consuming dead vole | consumption | NA |
| 46 | yes | 10-Jul-24 | UnID juvenile |  | P | Juvenile eating vole remnants | consumption | NA |
| 47 | yes | 10-Jul-24 | Amazon | F | A | Amazon consuming leftover dead vole at Tree J | consumption | NA |
| 48 | yes | 11-Jul-24 | LA | F | A | Consuming dead vole | consumption | NA |
| 49 | yes | 11-Jul-24 | 2 UnID adults  UnID juvenile |  | A  P | Unidentified adult chase, catch, kill, and consume vole at tree H. 8:42 Unmarked juvenile sat <1m from vole eater. 8:43 a second UnID adult steals vole from the original squirrel and began consuming it. 8:46 second adult goes into burrow without vole. | hunting, competition, consumption | yes |
| 50 | yes | 11-Jul-24 | bowtie | M | A | Bowtie unsuccessfully chases a vole. | hunting | no |
| 51 | yes | 11-Jul-24 | 2 UnID juveniles |  | P | 2 UnID juveniles simultaneously chase the same vole at Tree K. | hunting | no |
| 52 | yes | 11-Jul-24 | UnID adult |  | A | unID squirrel consumes vole remains at tree H | consumption | NA |
| 53 | yes | 11-Jul-24 | UnID juvenile |  | P | consuming dead vole | consumption | NA |
| 54 | yes | 11-Jul-24 | necktie |  | A | consuming dead vole | consumption | NA |
| 55 | yes | 11-Jul-24 | line_over_blot  UnID juvenile | M | P  P | line_over_blot consuming dead vole under gate near area A. UnID juvenile took vole and ran towards road/ area A with it. | competition, consumption | NA |
| 56 | yes | 11-Jul-24 | Capital_Z  barnacle | F  M | P  A | 11:34 at D_Area, Capital_Z eating a dead vole. Barnacle, keyhole and an unmarked juvenile are sitting <1m away. At 11:36 at D_Area, Barnacle seen eating dead vole from Capital_Z. Keyhole and UnID juvenile had left. Capital Z was still sitting <1m from barnacle as he consumed the vole. | competition, consumption | NA |
| 57 | yes | 12-Jul-24 | UnID juvenile |  | P | consuming dead vole | consumption | NA |
| 58 | yes | 12-Jul-24 | UnID adult |  | A | consuming dead vole | consumption | NA |
| 59 | yes | 12-Jul-24 | Aries  2 UnID juveniles | F | A  P | Aries consuming dead vole. Unmarked juvenile approaches and sits < 1m. 2nd juvenile approaches and greets Aries, sits < 1m. | consumption | NA |
| **#** | **Obs. day** | **Date** | **Fur mark** | **Sex** | **Age** | **Description** | **Activity** | **Successful hunt?** |
| 59 | yes | 12-Jul-24 | 2 UnID juvenile  Aries  asterisk | F  F | P  A  A | Unmarked juvenile 1 eating a piece of aries' vole, goes to aries for another piece and consumes it <1m away from aries, aries stops eating vole. 9:31 juvenile 1 takes the rest of the vole (about 1/2) and consumes it at a burrow entrance. Asterisk approaches and chases juvenile 1, juvenile 1 flees into burrow with vole and emerges after one minute to continue feeding on the vole. Juvenile 2 sits <1m. Unmarked juvenile 2 feeds on a piece of Aries' vole | consumption, competition | NA |
| 60 | yes | 12-Jul-24 | opera | F | P | Hunt, kill, and consume vole | hunting, consumption | yes |
| 61 | yes | 12-Jul-24 | California | M | A | unsuccessfully chasing vole | hunting | no |
| 62 | yes | 12-Jul-24 | UnID juvenile |  | P | unsuccessfully chasing live vole | hunting | no |
| 63 | yes | 12-Jul-24 | 2 UnID juveniles |  | PP | 2 unidentified juveniles simultaneously chase after the same vole (unsuccessfully) | hunting | no |
| 64 | no | 18-Jul-24 | roman_numeral_5 | M | P | Seen eating a vole near logs, ran across path into burrow in coyote with vole in mouth. | consumption | NA |
| 65 | yes | 19-Jul-24 | seventy_six | F | A | Unsuccessful vole chase | hunting | no |
| 66 | yes | 19-Jul-24 | Capital_Z | F | P | chase vole and pounce, unsuccessfully. | hunting | no |
| 67 | yes | 19-Jul-24 | Capital_Z | F | P | consuming vole remains | consumption | NA |
| 68 | yes | 19-Jul-24 | UnID juvenile |  | P | hunt, kill, and consume vole | hunting, consumption | yes |
| 69 | yes | 23-Jul-24 | dracula | F | A | consuming dead vole | consumption | NA |
| 70 | yes | 23-Jul-24 | Dracula  unID | F | A | 9:00 at Tree_R: Dracula seen chasing live vole; 9:14 at log_3: consuming dead vole; 10:56 at log_3: unID adult consuming dead vole from Dracula earlier | hunting, consumption | yes |
| 71 | yes | 24-Jul-24 | Ladder  SD | M  F | A  P | 10:47 at Tree_H and Area_A: Ladder carries dead vole from H tree to gate, starts feeding on vole, then carries it to Area_A where he continues consuming the vole. 11:23-11:29 at Area_A: SD eats remains of ladder's vole from earlier in the day, while ladder sit about 1.5 m away | consumption | NA |
| **#** | **Obs. day** | **Date** | **Fur mark** | **Sex** | **Age** | **Description** | **Activity** | **Successful hunt?** |
| 72 | yes | 24-Jul-24 | Capital_Z | F | P | Capital Z seen chasing, catching, killing, and consuming a vole at tree E at 11:08. At 11:10 she was scared off by a dog and carried the vole with her into burrow. | hunting, consumption | yes |
| 73 | yes | 25-Jul-24 | libra | M | P | consuming vole remains | consumption | NA |
| 74 | no | 30-Jul-24 | Capital_ Z  battery | F  F | P  A | 13:20 at Tree_C: Capital_Z briefly enters a burrow at Tree C, a squeal (presumably the vole) was heard, and capital_Z re-emerges about 2 s after with a dead vole in its mouth running over to tree I to start consuming it. Battery emerges from the same burrow at Tree C shortly after capital_Z, followed by an attempt to steal the vole; 13:23 at Tree_I: battery attempts to steal the vole. Capital_Z flees with vole in its mouth and disappears out of sight, battery following. | hunting, consumption, competition | yes |
